# Antimicrobial susceptibilities of *Neisseria gonorrhoeae* in Canada, 2022

**DOI:** 10.14745/ccdr.v51i04a03

**Published:** 2025-04-03

**Authors:** Pamela Sawatzky, Robyn Thorington, Norman Barairo, Brigitte Lefebvre, Mathew Diggle, Linda Hoang, Samir Patel, Paul Van Caessele, Jessica Minion, Guillaume Desnoyers, David Haldane, Xiaofeng Ding, Lillian Lourenco, Genevieve Gravel, Irene Martin

**Affiliations:** 1 National Microbiology Laboratory Branch, Public Health Agency of Canada, Winnipeg, MB; 2 Laboratoire de santé publique du Québec, Ste-Anne-de- Bellevue, QC; 3 Provincial Laboratory for Public Health, Edmonton, AB; 4 BC Centre for Disease Control Public Health Laboratory, Vancouver, BC; 5 Public Health Ontario Laboratory, Toronto, ON; 6 Cadham Provincial Laboratory, Winnipeg, MB; 7 Roy Romanow Provincial Laboratory, Regina, SK; 8 Dr. Georges-L.-Dumont University Hospital Centre, Moncton, NB; 9 Queen Elizabeth II Health Sciences Centre, Halifax, NS; 10 Queen Elizabeth Hospital, Charlottetown, PEI; 11 Centre for Communicable Diseases and Infection Control Branch, Public Health Agency of Canada, Ottawa, ON

**Keywords:** gonorrhea, *Neisseria gonorrhoeae*, antimicrobial resistance, antimicrobial susceptibility, national surveillance system, passive surveillance

## Abstract

**Background:**

In Canada, gonorrhea is the second most prevalent sexually transmitted bacterial infection. The Gonococcal Antimicrobial Surveillance Programme-Canada (GASP-Canada), a passive surveillance system monitoring antimicrobial resistance in *Neisseria gonorrhoeae* in Canada since 1985, is the source for this summary of demographics, antimicrobial resistance and *N. gonorrhoeae* multi-antigen sequence typing (NG-MAST) of gonococcal isolates collected in Canada in 2022.

**Objective:**

To summarize the antimicrobial resistance trends and molecular types of *N. gonorrhoeae* cultures in Canada from 2018 to 2022. These trends inform the federal, provincial and territorial guidelines for treatment of gonorrhea.

**Methods:**

Provincial and territorial public health laboratories submitted *N. gonorrhoeae* cultures and data to the National Microbiology Laboratory in Winnipeg as part of the surveillance system. The antimicrobial resistance and molecular type of each isolate received were determined.

**Results:**

In total, 3,855 *N. gonorrhoeae* cultures were identified and tested across Canada in 2022, a 12.1% increase since 2021 (n=3,439). Decreased susceptibility to cefixime declined in 2022 (0.3%) compared to 2018 (0.5%). Decreased susceptibility to ceftriaxone also declined between 2018 (0.6%) and 2022 (0.3%). Azithromycin resistance was similar in 2022 (8.1%) to what it was in 2018 (7.6%). In 2022, NG-MAST-17972 (13.3%) was the most prevalent sequence type in Canada.

**Conclusion:**

The spread of antimicrobial-resistant gonorrhea is a significant public health concern. The continued regional and national surveillance of antimicrobial resistance in *N. gonorrhoeae* is essential in ensuring effective treatment therapies are recommended.

## Introduction

In 2022, 35,956 cases of gonorrhea were reported in Canada, making it the second most reported sexually transmitted bacterial infection (STI) in the country. In 2022, the Canadian reported rate of gonorrhea was 92.3 per 100,000 population, which is more than double the 2013 rate of 40.56 per 100,000 population ((1)). Risk factors associated with gonorrhea may include sexual contact with a person who has gonorrhea, unprotected sex, age and a history of sexually transmitted and blood-borne infections ((2)). The causative agent, *Neisseria gonorrhoeae*, can infect not only the urogenital sites of both males and females, but also the throat, rectum and, if left untreated, blood and synovial fluid. While gonococcal infections are more commonly symptomatic in males, they are often asymptomatic in females, though they can be asymptomatic in both. Pharyngeal and rectal infections are more likely to be asymptomatic in all individuals ((2)). Pelvic inflammatory disease, infertility and disseminated gonococcal infections (DGI) causing arthritis, tenosynovitis and endocarditis are a few of the more serious complications that can arise from untreated gonorrheal infections ((3,4)).

Globally, the only available empiric treatment is the third-generation cephalosporin, ceftriaxone, used as either a monotherapy or as part of a dual therapy treatment, most often with azithromycin ((3)). Canada’s current treatment regimen recommended by the Public Health Agency of Canada is 250 mg of ceftriaxone intramuscularly or 800 mg of cefixime orally plus 1 g of azithromycin orally ((2)). The spread of ceftrixaone resistance and extremely drug-resistant (XDR-GC) is of concern globally as it threatens treatment.

In 2012, the World Health Organization (WHO) released a Global Action Plan with strategies to minimize the spread and impact of antimicrobial resistant *N. gonorrhoeae*. One of the suggested strategies is to set up national and international *N. gonorrhoeae* antimicrobial resistance (AMR) surveillance programmes ((3)). In 2009, WHO relaunched the global Gonococcal Antimicrobial Surveillance Programme (GASP) and the Enhanced GASP (EGASP) is currently expanding into new countries around the globe ((5)).

Since 1985, Canada’s passive national surveillance program, the Gonococcal Antimicrobial Surveillance Program-Canada (GASP-Canada) has been reporting trends of antimicrobial-resistant gonorrhea in Canada to ensure efficacy of current treatments and to inform federal treatment guidelines. Azithromycin resistance (AZI-R) in Canada has surpassed 5%, which is the benchmark suggested by the WHO for review of the use of an antimicrobial as a recommended treatment, and cases of cephalosporin-resistant *N. gonorrhoeae* were identified in Canada between 2017 and 2021 ((6–8)).

Isolates submitted to GASP-Canada undergo antimicrobial susceptibility testing (AST) and molecular characterization using *N. gonorrhoeae* multi-antigen sequence typing (NG-MAST). The NG-MAST typing shows a close association with AMR and can be used to investigate treatment failures and outbreaks due to its high level of discernment ((9–11)).

This report summarizes the AMR trends and molecular types of *N. gonorrhoeae* cultures from across Canada that were sent to GASP-Canada between 2018 and 2022. In addition to informing stakeholders from across the country of *N. gonorrhoeae* AMR trends within their jurisdiction, the data is used to inform treatment guidelines.

## Methods

### Surveillance

*Neisseria gonorrhoeae* cultures with resistance or decreased susceptibility to at least one antimicrobial are voluntarily sent to the National Microbiology Laboratory (NML) by provincial and territorial partners as part of GASP-Canada. Provincial and territorial laboratories that do not perform AST send all of their cultures for testing at NML. As of 2018, Alberta, British Columbia and Québec send AST and client data (in the form of minimum inhibitory concentrations [MICs] of isolates not sent to NML for testing and these MICs are included in our analysis. All resistant gonorrhea cultures from Alberta are sent to NML for testing. Québec and British Columbia send isolates to NML that either: 1) are resistant to azithromycin; 2) have decreased susceptibility to cefixime and/or ceftriaxone; or 3) are approaching resistance/decreased susceptibility to these antimicrobials. The AST and client data for the remaining isolates tested in Québec and British Columbia were submitted to NML. Nunavut and Yukon did not report or send any cultures to the NML between 2018 and 2022. The total number of *N. gonorrhoeae* cultures tested across Canada was used as the denominator in resistance calculations, unless otherwise noted.

### Isolate testing

Antimicrobial susceptibility testing using agar dilution (CLSI M100) and/or whole genome sequencing (WGS) methods ((9)) was performed on all *N. gonorrhoeae* cultures received by NML (n=2,544). Minimum inhibitory concentrations for seven antimicrobials were determined and interpretation of results was based on the Clinical and Laboratory Standards Institute for five of them (penicillin, tetracycline and azithromycin all resistant [R] when MIC is at least 2 mg/L; ciprofloxacin R when MIC is at least 1 mg/L; spectinomycin R when MIC is at least 128 mg/L) ((12)). The WHO guidelines were used for ceftriaxone (decreased susceptibility [DS] when MIC is at least 0.125 mg/L) and cefixime (DS when MIC is at least 0.25 mg/L) ((3)) (**Appendix**, Supplemental material Table S1). Testing of ß-lactamase was performed on all submitted cultures. Cultures with tetracycline MICs of at least 16 mg/L were tested for the *tetM* plasmid by polymerase chain reaction (PCR) ((13)) or with whole-genome sequence (WGS) data ((14)).

Multidrug-resistant *N. gonorrhoeae* (MDR-GC) is defined as a culture with either decreased susceptibility to the cephalosporins or resistance to azithromycin plus resistance to at least two other antimicrobials. Extensively drug-resistant *N. gonorrhoeae* (XDR-GC) is defined as resistance to azithromycin and decreased susceptibility/resistance to cephalosporins plus resistance to two other antimicrobials.

Genotyping of cultures was determined by NG-MAST using PCR ((11)) and/or WGS ((10)). SeqMan Pro 15 (DNAStar, Madison, Wisconsin) was used to assemble strands of Sanger-sequenced DNA and the sequence type (ST) was determined when sequences were submitted to the PubMLST *Neisseria* spp. database.

### Whole-genome sequencing

The DNA from isolates on which WGS was successfully performed (n=2,502) was prepared using the LuminUltra RNA Isolation Kit (LuminUltra, Fredericton, New Brunswick) and the E-Z 96™ Disruptor Plate C Plus (Omega BioTek, Nocross, Georgia). Briefly, the sequencing method used involved creating libraries (using Nextera sample preparation kits [Illumina, San Diego, California]) with 300 bp paired-end index reads generated on the Illumina NextSeq platform (Illumina). Galaxy Version 1.0.4+galaxy was used to assess the quality of the reads, assemble them, and analyze single nucleotide variants with NCCP1145 (GenBank accession number NC_011035) as the mapping reference. Whole-genome sequence data was used to detect molecular AMR markers and to determine NG-MAST STs ((10)).

### Data analysis

Age, sex, isolation site, province and date of collection were provided with the *N. gonorrhoeae* isolates. Duplicate isolates were identified and removed from the denominator if multiple isolates from the same client had the same ST and were collected within four weeks of each other. Once a potential set of duplicates was identified, a hierarchy of isolation sites was used to determine which of the isolates were considered the duplicates. The order of the hierarchy was 1) sterile site (DGI), 2) throat, 3) rectal and 4) urogenital. This hierarchy is based on morbidity of the infection (for DGI) and then the efficacy of treatment at an isolation site, with isolates collected from throat cultures having the lowest treatment efficacy and urogenital isolates having the highest.

Each figure includes the denominator used in its description. Trends of AMR and STs were determined nationally. Azithromycin resistance (AZI-R) and cefixime and ceftriaxone DS (CFM-DS and CRO-DS, respectively) were also analyzed at the provincial or territorial level. Correlation of the most common STs with AMR was also indicated. Comparisons of AMR proportions were made using the Fisher’s exact test, with a 99% confidence interval employing EpiCalc 2000 (version 1.02; Brixton Health).

## Results

### Isolates tested, demographics and isolation sites

**Figure 1:**
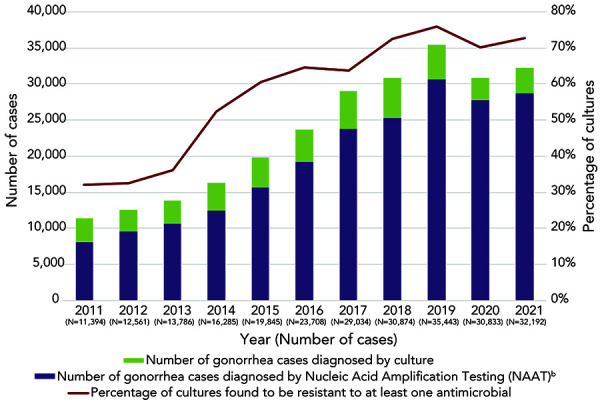
Reported *Neisseria gonorrhoeae* cases in Canada, 2011–2022^a,b^ Abbreviation: NAAT, nucleic acid amplification testing ^a^ Approximately 10% of all gonorrhea cases were diagnosed by culture in Canada in 2022. The rest was detected using nucleic acid amplification test technology ^b^ The number of gonorrhea cases diagnosed by nucleic acid amplification testing is determined by subtracting the number of cultures tested across Canada from the number of gonorrhea cases reported. Adopted from: Public Health Agency of Canada. Reported cases from 1924 to 2022 in Canada – Notifiable diseases on-line. Ottawa, ON: PHAC; 2022. https://diseases.canada.ca/notifiable/charts?c=pl

In 2022, *N. gonorrhoeae* isolates from 3,855 cases (Appendix, Supplemental material Table S2) from across Canada were tested. A provincial and territorial breakdown of the number of cultures collected by jurisdiction is found in Appendix, Supplemental material Table S3. Over 75% (75.5%, n=2,910/3,855) of the cultures were resistant to at least one antibiotic (**Figure 1**, Appendix, Supplemental material Table S2). Gonorrhea cases that were diagnosed using nucleic acid amplification tests (NAATs) are not included in this calculation. Antimicrobial susceptibility testing is not routinely performed on NAAT specimens, which accounted for 89% of diagnosed and reported gonorrhea cases in Canada in 2021 (Figure 1).

In 2022, the NML received data, including age, gender and isolation site for 3,393 cases. The majority of cases 70.9% (n=2,404) were from individuals aged 21–40 years old, with 20.5% (n=694) older than 40 years and 8.5% (n=288) younger than 21 years. Isolates from males dominated (83.7%, n=2,840) with 15.9% (n=540) from females; three (0.08%) were from gender diverse individuals and 10 (0.3%) did not specify. The penis/urethra was the prevalent isolation site among males (58.9%, n=1,673/2,840) and the throat among females (39.1%, n=211/540). See Appendix, Supplemental material Table S4 for more details.

### Cephalosporin antimicrobial trends in Canada, 2018–2022

**Figure 2:**
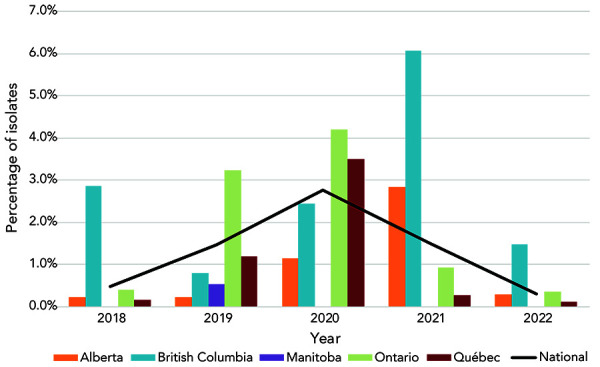
Percentage of *Neisseria gonorrhoeae* cultures with decreased susceptibility to cefixime by province, 2018–2022^a,b^ ^a^ Provinces included in this figure are only those that submitted at least one culture that had decreased susceptibility to cefixime to the National Microbiology Laboratory ^b^ Denominators used for the calculations of the percentages are the number of cultures tested in each province (Appendix, Supplemental material Table S4)

The proportion of CFM-DS (MIC of at least 0.25 mg/L) decreased significantly (*p*<0.001) from 2.8% in 2020 to 1.5% in 2021 and 0.3% (n=12) in 2022 (**Figure 2**).

**Figure 3:**
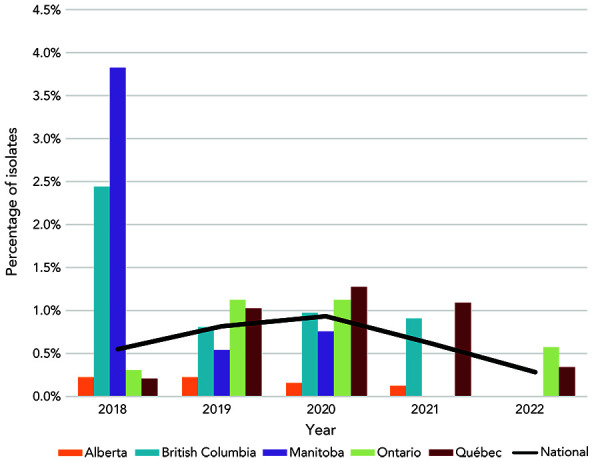
Percentage of *Neisseria gonorrhoeae* cultures with decreased susceptibility to ceftriaxone by province, 2018–2022^a,b^ ^a^ Provinces included in this figure are only those that submitted at least one culture that had decreased susceptibility to ceftriaxone to the National Microbiology Laboratory ^b^ Denominators used for the calculations of the percentages are the number of cultures tested in each province (Appendix, Supplemental material Table S4)

Decreased susceptibility to ceftriaxone (MIC of at least 0.125 mg/L) declined, although the decrease between years has not been significant (*p*>0.001), from 0.9% in 2020 to 0.6% in 2021 and 0.3% (n=11) in 2022 (**Figure 3**). There were no ceftriaxone-resistant (CRO-R) isolates identified in Canada in 2022.

### Azithromycin resistance in Canada, 2018–2022

**Figure 4:**
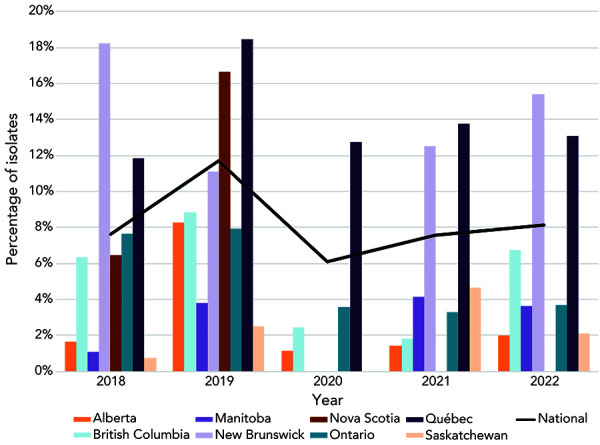
Percentage of azithromycin-resistant *Neisseria gonorrhoeae* cultures by province, 2018–2022^a,b^ ^a^ Provinces included in this figure are only those that submitted at least one culture that was azithromycin-resistant to the National Microbiology Laboratory. Newfoundland and Labrador had one azithromycin-resistant isolate in 2019 ^b^ Denominators used for the calculations of the percentages are the number of cultures tested in each province (Appendix, Supplemental material Table S4)

**Figure 5:**
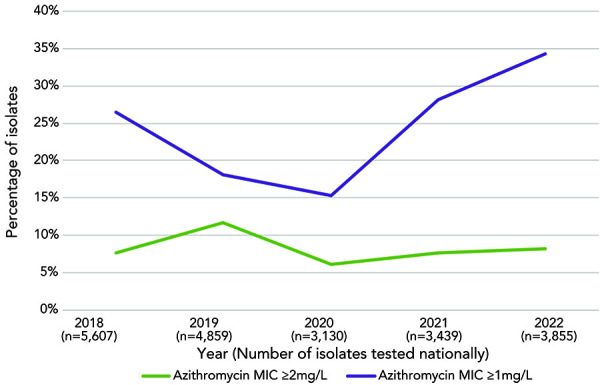
Trends of the percentage of azithromycin minimum inhibitory concentrations for *Neisseria gonorrhoeae* at greater than or equal to 1 mg/L and greater than or equal to 2 mg/L Abbreviation: MIC, minimum inhibitory concentration

The proportion of AZI-R in 2022 (8.1%, 313/3,855) was similar to that identified in 2018 (7.6%), 2020 (6.1%) and 2021 (7.6%) (**Figure 4**). The majority of AZI-R isolates across Canada were identified in Québec (77%, n=234/304), however, the highest AZI-R proportion within a jurisdiction was led by New Brunswick (15.4%, 6/39), followed by Québec (13.1%, n=234/1,785). The proportion of isolates within an azithromycin MIC of at least 1 mg/L increased (*p*<0.001), from 18.2% in 2019 and 15.3% in 2020 to 28.1% and 34.3% in 2021 and 2022, respectively (**Figure 5**). High-level azithromycin resistance (HL-AZI-R; AZI MIC of at least 512 mg/L) was identified in 11 isolates from 2022, primarily in British Columbia (n=8), with two in Québec and one in Ontario. These isolates were also resistant to ciprofloxacin, with three being resistant to tetracycline as well.

### Resistance trends in other antimicrobials, 2018–2022

**Figure 6:**
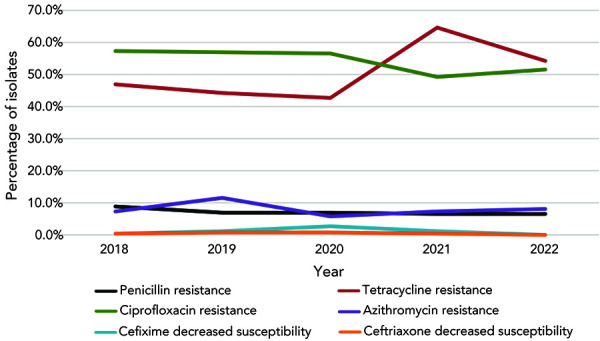
Percentage of antimicrobial resistance of *Neisseria gonorrhoeae* isolates tested in Canada, 2018–2022^a,b^ ^a^ Percentages are based on the total number of isolates tested nationally: 2018, n=5,607; 2019, n=4,859; 2020, n=3,130; 2021, n=3,439; 2022, n=3,855 ^b^ Due to some provinces not testing all seven antimicrobials from 2018 to 2022, penicillin denominators were 3,883, 3,822, 2,409, 2,334 and 3,329, respectively. In 2020, 2021 and 2022, tetracycline denominators were 2,409, 2,334 and 3,328, respectively

Resistance to ciprofloxacin (CIP-R, 51.7%) and tetracycline (TET-R, 54.4%) remained high among *N. gonorrhoeae* isolates in 2022. Provincially, CIP-R ranged from about 15%–65%; the approximate range for TET-R is 30%–60%. Penicillin resistance (PEN-R) has remained below 7% since 2020 (**Figure 6**).

Multidrug-resistant (MDR-GC) and extensively drug-resistant (XDR-GC) gonococci in Canada, 2018–2022

The proportion of MDR-GC cultures identified in 2022 (8.2%) was comparable to that found in 2021 (7.8%) and 2018 (8.0%). In 2019, there was a spike in MDR-GCs (12.4%) followed by a drop in 2020 (6.3%) (Appendix, Supplemental material Figure S1). One XDR-GC culture was identified in Canada in 2022 (0.03%), similar to the last three years (2019: 0.02%, n=1; 2020: 0.06%, n=2; and 2021: n=0) but a decrease from the number of XDR-GCs was identified in Canada in 2018 (0.12%, n=7). (Appendix, Supplemental material Figure S2, Table S5).

### *Neisseria gonorrhoeae* multi-antigen sequence typing trends in Canada

**Figure 7:**
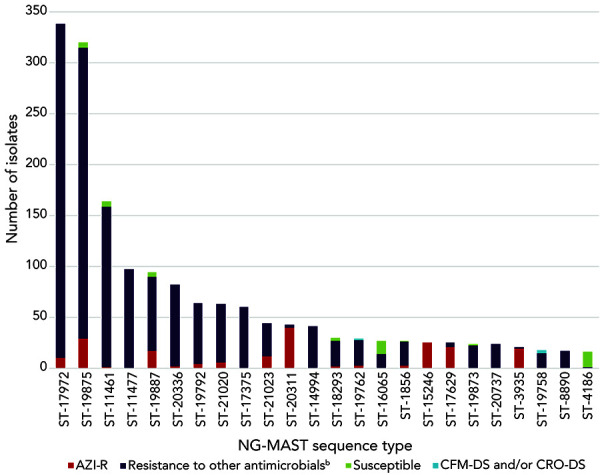
Distribution of antimicrobial resistance characterizations within *Neisseria gonorrhoeae*-multi-antigen sequence typing sequence types, 2022 (n=2,544)^a,b^ Abbreviations: AZI-R, azithromycin-resistant; CFM-DS, cefixime decreased susceptibility; CIP-R, ciprofloxacin-resistant; CRO-DS, ceftriaxone decreased susceptibility ^a^ Figure does not include 14 isolates that were non-typeable. This graph represents 1,694 isolates. The remaining 836 isolates are dispersed among 391 sequence types containing 1–15 isolates each ^b^ Other antimicrobials include ciprofloxacin, erythromycin (isolates with agar dilution results), penicillin and tetracycline

In 2022, 2,530 of the 2,544 cultures submitted were successfully typed for NG-MAST. The most frequently detected NG-MAST sequence type in Canada was ST-17972 (n=338), followed by ST-19875 (n=320) (**Figure 7**). All ST-17972 isolates were resistant to CIP and TET. Azithromycin resistance was identified in two of the isolates. Azithromycin resistance was higher in ST-19875 isolates (9.1%, n=29/320), although most were only TET-R (89.4%, n=286/320). No CFM-DS or CRO-DS were identified within these two STs.

Both ST-17972 and ST-19875 have quickly spread across Canada since they were first detected in 2019 and in 2020, respectively (Appendix, Supplemental material Figure S3). Four of the most prevalent STs in Figure 7 have strong associations with AZI-R, with all ST-15246 being resistant in 2022 (n=25/25), as well as 93.0% (n=40/43) of ST-20311, 90.5% (n=19/21) of ST-3935 and 84.0% (n=21/25) of ST-17629. The STs of the HL-AZI-R isolates are ST-20927 (n=7), ST-20904 (n=3) and ST-22036 (n=1). The primary NG-MAST of CFM-DS isolates was ST-19758 (25.0%, n=3/12). ST-19836 was the prevalent NG-MAST of CRO-DS isolates (27.3%, n=3/11).

## Discussion

The number of cases of gonorrhea reported in Canada in 2022 (n=35,956) increased compared to 2018 (n=30,874) ((1)). Unfortunately, the proportion of cases diagnosed by culture, as opposed to by NAAT, has decreased every year since 2011, when 30% of cases had cultures for testing. Since 2020, only 10% of reported cases included cultures for AST, which means that the AMR of all *N. gonorrhoeae* circulating in Canada is determined from fewer isolates.

Based on the cultures tested in 2022, the proportion of resistant *N. gonorrhoeae* identified in Canada continues to increase. The high level of resistance is primarily attributable to TET and CIP. Decreased susceptibility to the currently recommended treatment antimicrobials (CFM and CRO) has dropped to the lowest levels identified in over five years. Decreased susceptibility to CFM was identified in only four provinces in 2022, and for CRO, in only two provinces. Unlike 2021, no cephalosporin-resistant isolates were identified, with CRO and CFM MICs exceeding 0.125 mg/L and 0.25 mg/L, respectively.

Azithromycin resistance, however, has remained high. In the last seven years, the level of AZI-R in Canada has exceeded the 5% threshold set by WHO ((3)) for reviewing current treatment therapies, although provincially, rates of resistance have varied. The 11 HL-AZI-R isolates identified in 2022 represent the largest number of such isolates identified in one year in Canada, with five or less isolated per year in the last 10 years.

Like Canada, Australia also identified the highest number of HL-AZI-R *N. gonorrhoeae* per annum in 2022. Unlike Canada, however, their AZI-R proportion was 3.9% and has been stable since 2019. Their 2022 CRO-DS proportion was a little higher than Canada, at 0.51%, which includes eight CRO-R isolates. The majority of Australia recommends dual treatment therapy of CRO and AZI ((15)).

In Europe, 11% of *N. gonorrhoeae* isolates were identified with AZI MICs greater than 1 mg/L in 2020, 0.5% of isolates had cefixime MICs greater than 0.125 mg/L and one CRO-R isolate was identified ((16)). England saw an increase in decreased susceptibility to AZI (MICs greater than 0.5 mg/L), from 4.7% in 2016 to 8.7% in 2020, which led to a change in recommended therapy from CRO and AZI dual therapy to 1 g of CRO monotherapy in 2019. The increased CRO dosage (500 mg –1g) in the new recommended treatment may be responsible for the decrease in CRO-DS (MIC greater than 0.03 mg/L), from 7.1% in 2018 to 1.4% in 2020. Cefixime resistance has remained low at 0.6% ((17)).

The two highly related HL-AZI-R STs were first and primarily identified in British Columbia, but were also found in Ontario and Québec later in the year, possibly suggesting travel-associated spread. Other new and diverse STs may be formed when non-AZI-R *N. gonorrhoeae* obtain the mutations necessary to become AZI-R due to the pressure exerted by AZI being used to treat either gonorrhea or other infections.

## Limitations

Policies for culturing suspected gonorrhea infections differ between regions and NML receives isolates and associated data from provinces and territories at their discretion. Interpretation of results is limited not only by the number of the cultures tested but by the source of the cultures, as bias may be introduced based on the risk group of the client.

The GASP-Canada does not receive information on risk factors, sexual orientation or sexual behaviour, among other epidemiological data that are associated with the clients from whom the cultures are collected. To try and address this limitation, the ESAG was established in 2014, with six provinces and territories now submitting their enhanced epidemiological and treatment data for gonorrhea cases. This information is then paired with the GASP-Canada culture AST data to more fully examine which treatments are being used and how that relates to trends in *N. gonorrhoeae* AMR ((18)).

The large proportion of NAATs used to diagnose gonorrhea are not included when calculating resistance rate in Canada. This may cause a geographical bias, as many rural regions do not have the capacity to culture gonorrhea.

## Conclusion

Gonorrheal infections, left untreated or failing treatment, can lead to infertility, pelvic inflammatory disease and DGI, which can include dermatitis, arthritis and, in rare cases, endocarditis, meningitis or osteomyelitis ((19,20)).

Due to the ability of *N. gonorrhoeae* to develop antimicrobial resistance, resolving gonorrheal infections is becoming increasingly difficult. Combination therapy of CRO or CFM plus AZI has been the recommended treatment for gonorrhea infections in Canada since 2011 ((21,22)) in response to the increase in decreased susceptibility to cephalosporins ((20)). At the time of writing this paper, given the persistent AZI-R rate detected in some provinces, the nationally recommended gonorrhea therapies are under review.

Sustained surveillance of AMR trends in *N. gonorrhoeae* informs national treatment guidelines to ensure the most effective therapies are recommended. Additionally, the use of NG-MAST molecular typing allows for tracking of the spread of AMR across the country. AMR threats can be addressed by public health authorities when detected by ongoing surveillance.

## Appendix

Supplemental material is available upon request to the author: robyn.thorington@phac-aspc.gc.ca

## References

[1] Public Health Agency of Canada. Reported cases from 1924 to 2022 in Canada – Notifiable diseases on-line. Ottawa, ON: PHAC; 2022. https://diseases.canada.ca/notifiable/charts?c=pl

[2] Public Health Agency of Canada. Canadian Guidelines on Sexually Transmitted Infections. Ottawa, ON: PHAC; 2016. https://www.canada.ca/en/public-health/services/infectious-diseases/sexual-health-sexually-transmitted-infections/canadian-guidelines.html

[3] World Health Organization. Global Action Plan to control the spread and impact of antimicrobial resistance in Neisseria gonorrhoeae. Geneva, CH: WHO; 2012. https://www.who.int/publications/i/item/9789241503501

[4] Hook EW 3rd, Kirkcaldy RD. A Brief History of Evolving Diagnostics and Therapy for Gonorrhea: lessons Learned. Clin Infect Dis 2018;67(8):1294–9. 10.1093/cid/ciy271 29659749 PMC6452490

[5] Unemo M, Sánchez-Busó L, Golparian D, Jacobsson S, Shimuta K, Lan PT, Eyre DW, Cole M, Maatouk I, Wi T, Lahra MM. The novel 2024 WHO Neisseria gonorrhoeae reference strains for global quality assurance of laboratory investigations and superseded WHO N. gonorrhoeae reference strains-phenotypic, genetic and reference genome characterization. J Antimicrob Chemother 2024;79(8):1885–99. 10.1093/jac/dkae176 38889110 PMC11290888

[6] Lahra MM, Martin I, Demczuk W, Jennison AV, Lee KI, Nakayama SI, Lefebvre B, Longtin J, Ward A, Mulvey MR, Wi T, Ohnishi M, Whiley D. Cooperative recognition of internationally disseminated ceftriaxone-resistant Neisseria gonorrhoeae strain. Emerg Infect Dis 2018;24(4):735–40. 10.3201/eid2404.171873 29553335 PMC5875269

[7] Berenger BM, Demczuk W, Gratrix J, Pabbaraju K, Smyczek P, Martin I. Genetic characterization and enhanced surveillance of ceftriaxone-resistant Neisseria gonorrhoeae strain, Alberta, Canada, 2018. Emerg Infect Dis 2019;25(9):1660–7. 10.3201/eid2509.190407 31407661 PMC6711210

[8] Sawatzky P, Lefebvre B, Diggle M, Hoang L, Wong J, Patel S, Van Caessele P, Minion J, Garceau R, Jeffrey S, Haldane D, Lourenco L, Gravel G, Mulvey M, Martin I. Antimicrobial susceptibilities of *Neisseria gonorrhoeae* in Canada, 2021. Can Commun Dis Rep 2023;49(9):388–97. 10.14745/ccdr.v49i09a05 38463902 PMC10919915

[9] Mlynarczyk-Bonikowska B, Malejczyk M, Majewski S, Unemo M. Antibiotic resistance and NG-MAST sequence types of *Neisseria gonorrhoeae* isolates in Poland compared to the world. Postepy Dermatol Alergol 2018;35(6):346–551. 10.5114/ada.2018.79780 30618519 PMC6320495

[10] Sawatzky P, Demczuk W, Lefebvre B, Allen V, Diggle M, Hoang L, Van Caeseele P, Haldane D, Minion J, Mulvey MR, Martin I. Increasing Azithromycin Resistance in Neisseria gonorrhoeae Due to NG-MAST 12302 Clonal Spread in Canada, 2015 to 2018. Antimicrob Agents Chemother 2022;66(3):e0168821. 10.1128/aac.01688-21 34978884 PMC8923198

[11] Martin IM, Ison CA, Aanensen DM, Fenton KA, Spratt BG. Rapid sequence-based identification of gonococcal transmission clusters in a large metropolitan area. J Infect Dis 2004;189(8):1497–505. 10.1086/383047 15073688

[12] Clinical and Laboratory Standards Institute. Performance Standards for Antimicrobial Susceptibility Testing: 34th Edition M100-S32. Wayne, PA: CLSI; 2024.

[13] Carballo M, Ng LK, Dillon JR. Detection of the tetM determinant in Neisseria gonorrhoeae using a non-radioactively labelled oligonucleotide probe. Mol Cell Probes 1994;8(3):205–8. 10.1006/mcpr.1994.1028 7969193

[14] Demczuk W, Martin I, Sawatzky P, Allen V, Lefebvre B, Hoang L, Naidu P, Minion J, VanCaeseele P, Haldane D, Eyre DW, Mulvey MR. Equations to predict antimicrobial MICs in Neisseria gonorrhoeae using molecular antimicrobial resistance determinants. Antimicrob Agents Chemother 2020;64(3):1–11. 10.1128/AAC.02005-19 31871081 PMC7038236

[15] Lahra MM, Van Hal S, Hogan TR. Australian Gonococcal Surveillance Programme Annual Report, 2022. Commun Dis Intell (2018) 2023;47:47. 10.33321/cdi.2023.47.45 37817315

[16] European Centre for Disease Prevention and Control. Gonococcal antimicrobial susceptibility surveillance in the Europe Union/European Economic Area – Summary of results for 2020. Stockholm, SE: ECDC; 2022. https://www.ecdc.europa.eu/en/publications-data/gonococcal-antimicrobial-susceptibility-surveillance-2020

[17] Merrick R, Cole M, Pitt R, Enayat Q, Ivanov Z, Day M, Sun S, Sinka K, Woodford N, Mohammed H, Fifer H. Antimicrobial-resistant gonorrhoea: the national public health response, England, 2013 to 2020. Euro Surveill 2022;27(40):1–6. 10.2807/1560-7917.ES.2022.27.40.2200057 36205171 PMC9540523

[18] Public Health Agency of Canada. Report on the Enhanced Surveillance of Antimicrobial-Resistant Gonorrhea (ESAG): Results from 2018–2021. Ottawa, ON: PHAC; 2024. https://www.canada.ca/en/public-health/services/publications/diseases-conditions/enhanced-surveillance-antimicrobial-resistant-gonorrhea-esag-2018-2021.html

[19] Suzaki A, Hayashi K, Kosuge K, Soma M, Hayakawa S. Disseminated gonococcal infection in Japan: a case report and literature review. Intern Med 2011;50(18):2039–43. 10.2169/internalmedicine.50.5586 21921393

[20] Boodman C, MacKenzie L, Navarro C, Alexander DC, Wuerz T. Gonococcal endocarditis in a 54-year-old man with acute arthritis. CMAJ 2021;193(50):E1918–20. 10.1503/cmaj.211038 34930767 PMC8687512

[21] Public Health Agency of Canada. National Surveillance of Antimicrobial Susceptibilities of Neisseria gonorrhoeae Annual Summary 2013. Winnipeg, MB: PHAC; 2013. https://www.canada.ca/en/public-health/services/publications/drugs-health-products/national-surveillance-antimicrobial-susceptibilities-neisseria-gonorrhoeae-annual-summary-2013.html

[22] Public Health Agency of Canada. Important Notice – Public Health Information Update on the Treatment for Gonococcal Infection. Ottawa, ON: PHAC; 2011. https://www.canada.ca/en/public-health/services/infectious-diseases/sexual-health-sexually-transmitted-infections/canadian-guidelines/alerts/2011/important-notice-public-health-information-update-on-treatment-gonococcal-infection.html

